# Expression of calpain-like proteins and effects of calpain inhibitors on the growth rate of *Angomonas deanei* wild type and aposymbiotic strains

**DOI:** 10.1186/s12866-015-0519-0

**Published:** 2015-09-29

**Authors:** Simone Santiago Carvalho de Oliveira, Aline dos Santos Garcia-Gomes, Claudia Masini d’Avila-Levy, André Luis Souza dos Santos, Marta Helena Branquinha

**Affiliations:** Laboratório de Investigação de Peptidases, Departamento de Microbiologia Geral, Instituto de Microbiologia Paulo de Góes, Universidade Federal do Rio de Janeiro (UFRJ), Rio de Janeiro, Brazil; Laboratório de Estudos Integrados em Protozoologia, Coleção de Protozoários, Instituto Oswaldo Cruz, Fundação Oswaldo Cruz, Rio de Janeiro, Brazil; Laboratório de Microbiologia, Instituto Federal de Educação, Ciência e Tecnologia – Campus Rio de Janeiro, Rio de Janeiro, Brazil

**Keywords:** Trypanosomatidae, *Angomonas*, Endosymbiont, Peptidase, Calpain, Calpain-like proteins

## Abstract

**Background:**

*Angomonas deanei* is a trypanosomatid parasite of insects that has a bacterial endosymbiont, which supplies amino acids and other nutrients to its host. Bacterium loss induced by antibiotic treatment of the protozoan leads to an aposymbiotic strain with increased need for amino acids and results in increased production of extracellular peptidases. In this work, a more detailed examination of *A. deanei* was conducted to determine the effects of endosymbiont loss on the host calpain-like proteins (CALPs), followed by testing of different calpain inhibitors on parasite proliferation.

**Results:**

Western blotting showed the presence of different protein bands reactive to antibodies against calpain from *Drosophila melanogaster* (anti-Dm-calpain), lobster calpain (anti-CDPIIb) and cytoskeleton-associated calpain from *Trypanosoma brucei* (anti-CAP5.5), suggesting a possible modulation of CALPs influenced by the endosymbiont. In the cell-free culture supernatant of *A. deanei* wild type and aposymbiotic strains, a protein of 80 kDa cross-reacted with the anti-Dm-calpain antibody; however, no cross-reactivity was found with anti-CAP5.5 and anti-CDPIIb antibodies. A search in *A. deanei* genome for homologues of *D. melanogaster* calpain, *T. brucei* CAP5.5 and lobster CDPIIb calpain revealed the presence of hits with at least one calpain conserved domain and also with theoretical molecular mass consistent with the recognition by each antibody. No significant hit was observed in the endosymbiont genome, indicating that calpain molecules might be absent from the symbiont. Flow cytometry analysis of cells treated with the anti-calpain antibodies showed that a larger amount of reactive epitopes was located intracellularly. The reversible calpain inhibitor MDL28170 displayed a much higher efficacy in diminishing the growth of both strains compared to the non-competitive calpain inhibitor PD150606, while the irreversible calpain inhibitor V only marginally diminished the proliferation.

**Conclusions:**

Altogether, these results indicate that distinct calpain-like molecules are expressed by *A. deanei,* with a possible modulation in the expression influenced by the endosymbiont. In addition, treatment with MDL28170 affects the growth rate of both strains, as previously determined in the human pathogenic species *Leishmania amazonensis* and *Trypanosoma cruzi*, with whom *A. deanei* shares immunological and biochemical relationships.

## Background

Trypanosomatid parasites of insects, which are generally non-pathogenic to humans, develop in the digestive tract of their respective hosts and are transmitted by coprophagy or predation [1]. Up to now, seven insect trypanosomatids were found carrying a cytoplasmic bacterial endosymbiont [2, 3], including *Angomonas deanei*. This species*,* previously named as *Crithidia deanei* [4], is usually found in dipterans and hemipterans in the choanomastigote form but also as opistomorphs, differing from choanomastigotes in the positioning of the kinetoplast [4]. Interestingly, the endosymbiont affects the morphology and ultrastructure of the host protozoan [2, 5] and complements essential biochemical pathways, such as heme and amino acid metabolism [5, 6]. Conversely, the endosymbiont is supplied with a stable environment and nutrients. Antibiotic treatment induces the loss of the bacterium, leading to an aposymbiotic strain. The maintenance of the aposymbiotic strain in laboratory is only possible with medium supplementation of essential components, such as heme and amino acids [5].

Our group has demonstrated that both strains displayed two extracellular peptidase classes: cysteine- and metallo-peptidase, being the latter more abundant in the aposymbiotic strain [7]. These results provided evidence that in *A. deanei*, and possibly in the other symbiont-harboring trypanosomatids, the presence of the symbiotic bacterium may diminish the secretion of proteolytic enzymes, since the symbiont supplies the host with either finished forms of amino acids or usable intermediates [6]. Both extracellular enzymes were later purified [8, 9], and the cysteine peptidase displayed common features with neutral, calcium-dependent cysteine peptidases, also known as calpains, such as the maximum activity at pH 7.0 in the presence of calcium and the complete blockage of its proteolytic activity by the cysteine peptidase inhibitor E-64 as well as by the calcium chelator EGTA [9]. This extracellular cysteine peptidase also showed cross-reactivity with the antibody against *Drosophila melanogaster* calpain (anti-Dm-calpain) and no cross-reactivity with anti-human calpain antibodies [9].

Calpains form one of the most important proteolytic systems of mammalian cells. The family of mammalian calpains contains 16 genes: 14 are protein-coding domains that contain cysteine peptidases, while the other two genes encode smaller, regulatory proteins that are associated with the catalytic subunit, such that these enzymes are heterodimeric proteins formed by a catalytic subunit of 80 kDa and a regulatory subunit of 27 kDa [10]. Numerous functions have been postulated for calpains in the human body with links to signal transduction, cell motility, cell cycle and apoptosis [10–12]. Calpain-like proteins (CALPs) differ in amino acid composition within the catalytic triad and the lack of similarities to the calcium-binding EF-hand-containing motifs found in calpains [10, 12]. In this sense, CALPs have been identified in mammals but mainly in invertebrates and in lower eukaryotes, such as fungi, protists, nematodes, plants and invertebrates [10]. A large and diverse family of CALPs was detected in trypanosomatids [13, 14], including *A. deanei* genome [15]. In these protozoa, CALPs were categorized into five groups, based on their structural features, but the absence of amino acid residues essential for catalytic activity and the moderate overall degree of sequence identity with human calpains suggest that most of these CALPs do not have proteolytic activity [13].

Further studies from our group using immunoblotting analysis showed that the anti-Dm-calpain antibody strongly recognized a polypeptide of approximately 80 kDa in *Leishmania amazonensis* promastigotes [16] as well as in *Trypanosoma cruzi* epimastigotes [17, 18]. In these studies, the calpain inhibitor MDL28170, which is a potent and cell-permeable calpain inhibitor, was added to replicating forms in different concentrations, and our results showed that it arrested the growth of both parasites, *L. amazonensis* and *T. cruzi*, in a dose-dependent manner [16, 17].

Altogether, these findings offered some important approaches for CALPs research in trypanosomatids: the detection of distinct CALPs by the usage of anti-calpain antibodies from different origins and with distinct specificities, the possible role of an endosymbiotic bacterium on the expression of these molecules as well as the ability of different calpain inhibitors with varying specificity to interfere with parasite proliferation. In this study, these tasks were performed with *A. deanei* wild type and aposymbiotic strains.

## Methods

### Parasites and cultivation

The wild type and aposymbiotic strains of *Angomonas deanei* were kindly supplied by Dr. Maria Cristina M. Motta (Instituto de Biofísica Carlos Chagas Filho, UFRJ, Brazil) and are deposited at Fiocruz Protozoa Collection under the accession numbers COLPROT 044 and COLPROT 248, respectively. Parasites were cultivated in 3.7 % (w/v) brain heart infusion medium supplemented with 0.002 % (w/v) hemin and 5 % (v/v) heat-inactivated fetal bovine serum for 48 h at 28 °C to reach log phase growth.

### Identification of CALPs in *A. deanei* wild type and aposymbiotic strains by Western blotting

*A. deanei* wild type and aposymbiotic strains (2 × 10^8^ cells) were collected in log growth phase by centrifugation at 3000 *g* for 5 min at 4 °C, washed three times with cold PBS and lysed with 100 μl of sodium dodecyl sulfate-containing polyacrylamide gel electrophoresis (SDS-PAGE) sample buffer (62 mM Tris–HCl, pH 6.8, 2 % SDS, 25 % glycerol, 0.01 % bromophenol blue and 1 mM β-mercaptoethanol). In order to obtain the spent culture medium, cells grown until the log phase were centrifuged in the same conditions described above under sterile conditions. For each 10^8^ cells present in the medium, 1 ml of sterile PBS was added. After incubation for 4 h at 28 °C, the suspension was centrifuged and the supernatant collected, filtered through a sterile membrane (0.22 μm) and concentrated by dialysis ("molecular mass cut off" 9000 Da) against polyethylene glycol 6000 overnight at 4 °C until 500 μl. SDS-PAGE sample buffer was added in a 7:3 ratio (conditioned supernatant:buffer, v/v). The viability of cells during incubation in PBS was verified by the absence of malate dehydrogenase, an intracellular enzyme, in the supernatant, as previously described [9].

Immunoblot analysis was performed with total cellular extracts (equivalent to 5 × 10^6^ cells) and the conditioned supernatant (equivalent to 10^8^ cells), as previously described [19]. Proteins were separated in 10 % SDS-PAGE under reducing conditions and the polypeptides were electrophoretically transferred at 4 °C at 100 V/300 mA for 2 h to nitrocellulose membranes. The membrane was blocked in 10 % low-fat dried milk dissolved in TBS (150 mM NaCl; 10 mM Tris, pH 7.4) containing 0.05 % Tween 20 (TBS/Tween) for 1 h at room temperature. Membranes were washed three times (10 min each) with the blocking solution and incubated for 2 h with a 1:500 dilution of the following rabbit antibodies: anti-Dm-calpain (polyclonal, raised against the 70-kDa C-terminal region of calpain from *Drosophila melanogaster* and kindly donated by Dr Yasufumi Emori – Department of Biophysics and Biochemistry, Faculty of Sciences, University of Tokyo, Japan) [20]; anti-CAP5.5 (monoclonal, raised against the cytoskeleton-associated protein from *Trypanosoma brucei* and kindly provided by Dr. Keith Gull – Sir William Dunn School of Pathology, University of Oxford, England) [21]; and anti-CDPIIb (polyclonal, raised against *Homarus americanus* calpains and kindly donated by Dr. Donald L. Mykles – Colorado State University, USA) [22]. The secondary antibody used was peroxidase-conjugated goat anti-rabbit immunoglobulin G at 1:25,000 followed by chemiluminescence immunodetection after reaction with ECL reagents. An anti-α-tubulin monoclonal antibody produced in rabbit (Sigma) at 1:500 dilution was also used as a control for sample loading in the immunoblot. The relative molecular mass of the reactive polypeptides was calculated by comparison with the mobility of SDS-PAGE standards and the densitometric analysis was performed using the ImageJ program.

### Sequence data analysis

A search for Dm-calpain, CAP5.5 and CDPIIb homologous proteins in *A. deanei* was conducted using the BlastP algorithm and the nr database at NCBI (GenBank). The following queries were compared in a BlastP analysis against *A. deanei* proteins found in GenBank database: the fragment of the AHN56408.1 protein used to generate the Dm-calpain antibody [20], AAG48626.1 protein for *T. brucei* anti-CAP5.5 calpain and AAM88579.1 for anti-CDPIIb lobster calpain. The theoretical molecular masses of homologous proteins were calculated using the ExPASy Server facilities (http://www.expasy.org). Identification of conserved domains was performed using the CDD tool at NCBI [23]. Prediction of palmitoylation and myristoylation sites was conducted using CSS-Palm 4.0 [24] and NMT available on ExPASy.

### Identification of CALPs in *A. deanei* wild type and aposymbiotic strains by flow cytometry

Wild type and aposymbiotic strains of *A. deanei* (5 × 10^6^ cells) were processed for flow cytometry analyses [17]. Briefly, parasite cells were fixed for 15 min in 0.4 % paraformaldehyde in phosphate-buffered saline (PBS: 150 mM NaCl, 20 mM phosphate buffer, pH 7.2) at room temperature, followed by extensive washing in the same buffer. Alternatively, the fixed cells were permeabilized by 0.01 % Triton X-100 in PBS for 15 min at room temperature and then washed twice in PBS. The fixed and permeabilized cells maintained their morphological integrity, as verified by optical microscopic observation. Cells were then incubated for 1 h at room temperature with a 1:100 dilution of the anti-Dm-calpain, anti-CAP5.5 and anti-CDPIIb antibodies. Cells were then incubated for an additional hour with a 1:100 dilution of fluorescein isothiocyanate (FITC)-labeled goat anti-rabbit IgG (Sigma). Finally, cells were washed three times in PBS and analyzed in a flow cytometry (FACSCalibur, BD Bioscience, USA) equipped with a 15 mW argon laser emitting at 488 nm. Non-treated cells and those treated with the secondary antibody alone were run in parallel as controls.

### Effects of calpain inhibitors on the growth rate of the parasites

The action of three cell-permeable calpain inhibitors was evaluated upon the growth rate of *A. deanei* wild type and aposymbiotic strains: MDL28170 (a reversible and competitive peptidomimetic inhibitor, also known as calpain inhibitor III; Z-Val-Phe-CHO; Z = *N*-benzyloxycarbonyl); calpain inhibitor V (an irreversible and competitive peptidomimetic inhibitor; Mu-Val-HPh-FMK; Mu = morpholinoureidyl; HPh = homophenylalanyl; FMK = fluoromethylketone); and PD150606 (3-(4-iodophenyl)-2-mercapto-(Z)-2-propenoic acid, a non-competitive calpain inhibitor directed towards the calcium-binding sites of calpain). These compounds (Calbiochem, San Diego, CA, USA) were dissolved in dimethylsulfoxide (DMSO) at 5 mM.

Briefly, parasites were counted using a Neubauer chamber and re-suspended in fresh medium to a final concentration of 10^6^ viable cells per milliliter. Viability was assessed by mobility and lack of staining after challenging with trypan blue [16]. Each calpain inhibitor was added to the culture medium at the concentrations indicated in the text to each experiment, and a dilution of DMSO corresponding to that used to prepare the highest drug concentration was assessed in parallel. After 24, 48, 72 and 96 h incubation at 28 °C, the number of viable, motile trypanosomatids was quantified by counting the flagellates in a Neubauer chamber. Alternatively, parasites grown for 48 h in the presence of the calpain inhibitors were washed five times in cold PBS (pH 7.2) prior to re-suspension in a drug-free fresh medium and allowed to grow for another 48 h, in order to evaluate the cidal or static effect. The 50 % inhibitory concentration (IC_50_) was evaluated after 48 h. This value was determined by linear regression analysis, by plotting the number of viable cells versus log drug concentration by use of Origin Pro 7.5 computer software.

### Statistical analysis

All experiments were carried out at least three times. Data were analyzed by Student’s *t*-test using EPI-INFO computer software. *P* values of 0.05 or less were considered statistically significant.

## Results and discussion

### Detection of CALPs in *A. deanei* wild type and aposymbiotic strains by Western blotting and genomic analysis

**Fig. 1:**
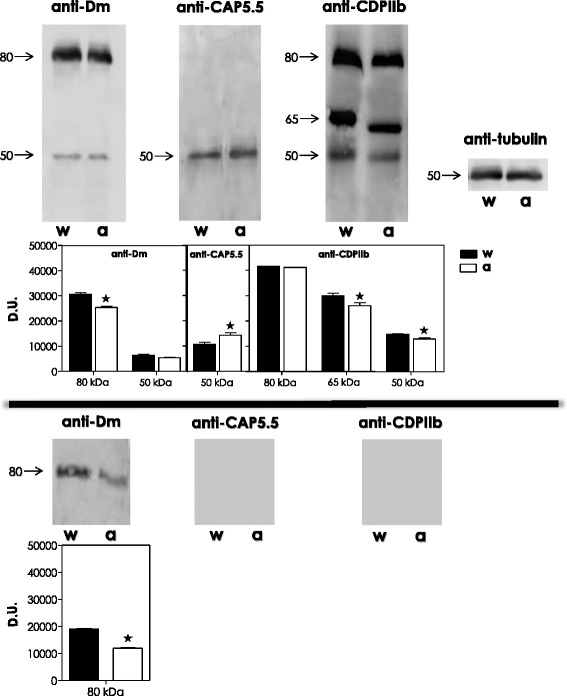
Detection of cross-reactivity between calpain-like proteins (CALPs) from *Angomonas deanei* wild type *(w)* and aposymbiotic *(a)* strains and anti-calpain antibodies by Western blotting. CALPs recognized in the whole cellular extract *(upper panel)* and in the conditioned supernatant *(lower panel)* by the anti-Dm-calpain, anti-CAP5.5 and anti-CDPIIb antibodies in each strain are shown. Anti-α-tubulin monoclonal antibody was used as a control for sample loading in the blots, revealing a protein band of 50 kDa in similar amount in both strains. The apparent molecular masses, expressed in kilodaltons, of each detected band are shown, and the densitometric analysis of the reactive bands is expressed as densitometric units (D.U.). The results represent means standard deviation of three independent experiments, and the asterisks denote statistic difference between wild type and aposymbiotic strains (*P* <0.05)

In this set of experiments, we aimed to evidence the CALPs expressed in *A. deanei* wild type and aposymbiotic strains by Western blotting assay using three anti-calpain antibodies from distinct origins and with different specificities. In this case, the same polypeptide bands were detected in both strains for each antibody tested (Fig. 1). Anti-Dm-calpain antibody reacted with two polypeptide bands with apparent molecular masses of 80 kDa and 50 kDa. When anti-CAP5.5 antibody was used, a single 50-kDa polypeptide was detected. The most complex profile was found with anti-CDPIIb antibody, for which three bands at 80 kDa, 65 kDa and 50 kDa were detected (Fig. 1).

An interesting observation detected here is that the anti-CAP5.5 monoclonal antibody reacted exclusively with a 50-kDa protein in both strains (Fig. 1). CAP5.5 protein was the first studied member of calpain-related genes in a trypanosomatid, specifically in *T. brucei*. This protein is characterized by the similarity to the catalytic region of calpain-type peptidases and it is detected exclusively in procyclic forms of *T. brucei*. On Western blotting, the protein was detectable as a single band of approximately 120 kDa in the cytoskeletal fraction of Triton X-100-extracted *T. brucei* cells. CAP5.5 has been shown to be both myristoylated and palmitoylated, suggesting a stable interaction with the cell membrane through interactions with the underlying microtubule cytoskeleton as well [21]. Another aspect that deserves consideration in the results presented herein is the higher expression of this protein in the aposymbiotic strain, as visualized in the Western blotting analysis, in comparison to its detection in the wild type strain (Fig. 1). This result was confirmed by the densitometric analysis, in which a significant increase of 30 % was detected in the aposymbiotic strain (Fig. 1).

It was also interesting to observe that a polypeptide band migrating at 80 kDa was detected in *A. deanei* wild type and aposymbiotic strains by cross-reactivity with the anti-Dm-calpain antibody (Fig. 1). This protein band was significantly more intense in the wild type strain (23 %) than the same band in the aposymbiotic strain, as confirmed by the densitometric analysis (Fig. 1). A polypeptide with 80 kDa was also recognized by anti-Dm-calpain antibody in *L. amazonensis* promastigote forms [16], *T. cruzi* clone Dm28c [17] and Y strain [18] epimastigote forms and in the insect trypanosomatid *Herpetomonas samuelpessoai* promastigote and paramastigote forms [25]. The detection of the same protein band in trypanosomatids from different genera may suggest that these parasites share the same antigen with invertebrate calpain-related enzymes. In this sense, our group has previously shown that some insect trypanosomatid proteins display immunological cross-reactivity with leishmanial gp63 metallopeptidase and *T. cruzi* cruzipain cysteine peptidase [19, 26], two well-known virulence factors present in these pathogenic trypanosomatids. This reflects the similarities in the basic cellular machinery between insect trypanosomatids and human pathogens, such as *T. brucei*, *T. cruzi* and *Leishmania* spp. [27].

Interestingly, the anti-CDPIIb antibody also reacted with an 80-kDa protein band in both strains (Fig. 1), and it was previously determined the immunological relationship between lobster CDPIIb and Dm-calpain [22], since anti-CDPIIb antibody detected Dm-calpain and proteolyzed fragments; conversely, anti-Dm-calpain antibody cross-reacted with CDPIIb. In addition, the 65-kDa and the 50-kDa bands in both strains that cross-reacted with the anti-CDPIIb antibody had their expression significantly decreased in the aposymbiotic strain by approximately 14 % and 17 %, respectively (Fig. 1).

The differential expression of the 80-kDa protein cross-reactive to anti-Dm-calpain antibody, the 50-kDa protein cross-reactive to anti-CAP5.5 antibody and both the 65-kDa and 50-kDa proteins cross-reactive to anti-CDPIIb antibody in each strain highlights the possible expression of some CALPs being modulated by the presence of the endosymbiont. Since previous studies showed that the symbiont interferes with several aspects of the host trypanosomatid metabolism, including the expression of different molecules [5, 19], the results presented herein suggest the possible involvement of the symbiont in the different expression of some CALPs in wild type and aposymbiotic strains of *A. deanei*. Intriguingly, the Western blotting analysis of the cell-free culture supernatant of *A. deanei* wild type and aposymbiotic strains showed a cross-reactive protein band at 80 kDa with the anti-Dm-calpain antibody, with a significant 40 % reduction in the amount of this polypeptide in the aposymbiotic strain (Fig. 1). However, no cross-reactivity was found with anti-CAP5.5 and anti-CDPIIb antibodies (Fig. 1). This band is possibly the cysteine peptidase detected previously by our group displaying proteolytic activity in *A. deanei* culture supernatant [9]. This enzyme was purified and migrated in SDS-PAGE as a single band of 80 kDa that cross-reacted with anti-Dm-calpain antibody, with optimum activity at neutral pH in the presence of calcium, sharing some features with calpains [9]. The detection of a higher amount of this CALP in the spent culture medium of the wild type strain corroborates the higher amount also detected in the whole cellular extracts in the Western blotting (Fig. 1). These data reinforce the differential expression of the 80-kDa protein in each strain as a result of the influence mediated by the presence of the endosymbiont.

Since we cannot exclude the possibility that the three antibodies used in this work may cross-react with proteins unrelated to CALPs in *A. deanei*, we performed a search in *A. deanei* genome for homologues of *D. melanogaster* calpain, *T. brucei* CAP5.5 and lobster CDPIIb calpain. All hits corresponding to homologues with e-value ranging from 1e-180 to 1e-05 corresponding to calcium-dependent cysteine peptidases had their theoretical molecular masses determined (data not shown). Among these hits, we selected those that presented molecular masses compatible with the cross-reactive protein bands detected in the Western blotting analysis. These hits were also investigated for palmitoylation and myristoylation sites, post-translational modifications that induce the addition of 238 Da and 210 Da to global protein mass, respectively.

For *D. melanogaster* calpain, five and two homologues presented molecular masses around 80 kDa and 50 kDa, respectively. For *T. brucei* CAP5.5, two homologues presented a molecular mass around 50 kDa, while for lobster CDPIIb calpain, five, one and three homologues presented molecular masses around 80 kDa, 65 kDa and 50 kDa, respectively (Table 1). Many of the predicted proteins included in Table 1 are likely to be myristoylated and/or palmitoylated. Acylation modification motif is a common feature shared by many CALPs in trypanosomatids, and it is most likely involved in the association with cellular membranes, as previously described for *T. brucei* CAP5.5 protein [13, 21].

**Table 1:** Possible *Angomonas deanei* calpain-like proteins recognized by the anti-calpain antibodies used in the present work.

Anti-calpain Antibody	Query protein accession #	Homologues in *Angomonas deanei*						
		Accession #	% Query cover	% Identity	E-value	Theoretical molecular mass (kDa)	Conserved domains ^a^	PTM (number of sites) ^b^
Anti-Dm-calpain	AHN56408.1	EPY32139.1	59	26	3e-39	80.2	cd00044	P(1)
							cl07679	
		EPY35937.1	59	26	4e-39	80.3	cd00044	P(1)
							cl07679	
		EPY35473.1	43	26	7e-31	81.0	cd00044	P(4) M(1)
							pfam09149	
		EPY17791.1	29	31	1e-22	80.7	cd00044	P(1) M(1)
							cl07679	
		EPY29841.1	37	25	3e-17	80.1	cd00044	P(1) M(1)
							pfam09149	
		EPY33036.1	26	29	4e-22	47.6	cd00051	-
		EPY17550.1	9	33	1e-05	55.0	cd00051	M (1)
Anti-CAP5.5	AAG48626.1	EPY42129.1	45	40	6e-77	45.7	cd00051	P (1) M (1)
							pfam09149	
		EPY33036.1	42	28	4e-47	47.6	cd00051	-
Anti-CDPIIb	AAM88579.1	EPY35937.1	49	31	1e-34	80.3	cd00044	-
							cl07679	
		EPY32139.1	49	31	1e-34	80.1	cd00044	-
							cl07679	
		EPY17791.1	57	28	1e-22	80.1	cd00044	P(1) M(1)
							pfam09149	
		EPY35473.1	53	27	7e-25	81.0	cd00044	P(4)
							pfam09149	
		EPY29841.1	19	29	1e-10	80.8	cd00044	P(1) M(1)
							pfam09149	
		EPY36766.1	34	23	4e-05	62.0	cd00051	P(1)
							pfam09149	
		EPY33036.1	30	28	4e-16	47.6	cd00051	-
		EPY42129.1	19	29	6e-11	45.7	cd00051	P(1) M(1)
							pfam09149	
		EPY17550.1	12	38	5e-05	55.0	cd00051	P(6)

^a^Conserved calpain domains: cd00044 (or CysPc domain) corresponds to the domains IIa and IIb of the catalytic site of calpains; cd00051 (or EFh domain) is a EF-hand, calcium-binding motif present in calpains; pfam09149 and cl07679 (DUF1935 superfamily) are domains found in hypothetical proteins and calpains, with unknown function. ^b^Number of possible post-translacional modification sites (PTM) for palmitoylation (P) and myristoylation (M)

At least one out of four conserved calpain domains (cd00044, cd00051, pfam09149 and cl07679) is presented in each homologue sequence (Table 1). In this sense, cd00044 (or CysPc domain) corresponds to the domains IIa and IIb of the catalytic site of calpains; cd00051 (or EFh domain) is a EF-hand, calcium-binding motif present in calpains; whereas pfam09149 and cl07679 (DUF1935 superfamily) are domains found in hypothetical proteins and calpains, with unknown function. The presence of at least one calpain conserved domain as well as the theoretical molecular mass are consistent with the recognition by each antibody tested (Fig. 1 and Table 1). In some cases, the high sequence similarity among these proteins indicates the possibility of being isoenzymes. Interestingly, the same homologue, EPY33036.1, can be recognized by the three antibodies, displaying a theoretical molecular mass of 47.6 kDa, which is consistent with the detection of a 50-kDa protein band that cross-reacted with these antibodies in the Western blotting analysis (Fig. 1). In addition, the five homologues presenting molecular masses around 80 kDa can be also recognized both by anti-Dm-calpain and anti-CDPIIb antibodies, which reinforces the immunological similarities between the proteins recognized by these antibodies (Fig. 1).

To provide another piece of evidence that the calpains identified by the antibodies used here were from the trypanosomatid and not from the symbiont, we performed a search using "calpain" and “cysteine peptidase” as queries on the nr database of the predicted proteins of *Candidatus Kinetoplastibacterium crithidii* at NCBI (GenBank). We also used all the calpain sequences from *Angomonas deanei*, and the peptide sequences used to produce the antibodies, as queries for a BlastP analysis using the same database. In all cases, no significant hit was observed, indicating that calpain molecules might be absent from the symbiont.

### Detection of CALPs in *A. deanei* wild type and aposymbiotic strains by flow cytometry

Flow cytometry analysis of *A. deanei* wild type and aposymbiotic strains was performed using the same set of anti-calpain antibodies employed in the Western blotting analysis (Table 2). In this analysis, the binding of the three anti-calpain antibodies was significantly enhanced when fixed cells were permeabilized, particularly for anti-Dm-calpain antibody (Table 2), which indicates that CALPs are located mainly in intracellular compartments but also in low levels on the cell surface. After Triton X-100 permeabilization, a similar percentage of fluorescent cells, for both strains, were detected for anti-Dm-calpain and anti-CDPIIb, with approximately 80 % of parasites labeled with the former and 40 % with the latter antibody. However, for aposymbiotic strain permeabilized cells, anti-CAP5.5 antibody bound two-fold higher (44 %) when compared with the binding to wild type strain (22 %). This fact may be correlated to the higher expression of the 50-kDa protein cross-reactive to this antibody in the aposymbiotic strain, as detected in Western blotting analysis (Fig. 1).

**Table 2:** Detection of cross-reactivity between calpain-like proteins from *Angomonas deanei* wild type and aposymbiotic strains and anti-calpain antibodies by flow cytometric analysis

Anti-calpain Antibody	Non-Permeabilized Parasites				Triton X-100-Permeabilized Parasites			
	Wild Type Strain		Aposymbiotic Strain		Wild Type Strain		Aposymbiotic Strain	
	% FC^a^	MFI^a^	% FC	MFI	% FC	MFI	% FC	MFI
Anti-Dm-calpain	6.3 ± 0.4	4.1 ± 0.2	5.3 ± 0.5	4.6 ± 0.5	80.0 ± 3.9	44.4 ± 6.8**	81.1 ± 8.9	19.7 ± 3.2**
Anti-CAP5.5	8.2 ± 0.8	4.7 ± 0.3	6.0 ± 1.0	4.3 ± 0.5	22.1 ± 1.8*	10.1 ± 0.2	44.2 ± 9.9*	13.5 ± 0.6
Anti-CDPIIb	6.1 ± 0.9	2.8 ± 0.2	4.4 ± 1.2	2.4 ± 0.2	39.8 ± 4.0	16.9 ± 1.5	39.9 ± 5.4	18.6 ± 0.4

^a^ % FC, percentage of fluorescent cells; MFI, mean of fluorescence intensity. Symbols (*,**) denote significant different concerning either the % FC (*) or MFI (**) between wild type and aposymbiotic strains (*P* <0.05). Additionally, both the percentage of antibody-labeled cells and the MFI values for all tested antibodies were significantly different between non-permeabilized and Triton X-100-permeabilized parasite cells. Representative data of the analysis of 10,000 cells from 3 experiments are shown

The flow cytometric analysis using the same anti-calpain antibodies also provided measurements for the relative levels of intracellular and surface CALPs expression in *A. deanei* wild type and aposymbiotic strains. As expected by the percentage of fluorescent cells detected, the permeabilization with Triton X-100 also raised significantly the mean of fluorescence intensity (MFI) values for the three anti-calpain antibodies used (Table 2). In non-permeabilized cells, the MFI levels to the same antibody were similar in wild type and aposymbiotic strains (Table 2). A similar labeling was also found for both strains when anti-CDPIIb and anti-CAP5.5 antibodies were used in permeabilized cells: for the former, MFI values varied from 16.9 in the wild type strain to 18.6 in the aposymbiotic strain, while for the latter MFI values detected were 10.1 and, 13.5 respectively (Table 2). In this sense, the presence of CALPs that cross-react with anti-CAP5.5 antibody was already observed in *T. cruzi* epimastigote forms by flow cytometry on the cell surface [17,18] but mainly in the intracellular milieu [17], in a similar pattern of distribution detected in the present work for *A. deanei* strains. Significantly higher MFI levels for intracellular CALPs by the usage of anti-Dm-calpain antibody were found for the wild type strain (MFI value 44.4) in comparison to the aposymbiotic strain (MFI value 19.7) (Table 2). This result corroborates the higher expression of the 80-kDa protein cross-reactive to this antibody in the wild type strain through the Western blotting analysis (Fig. 1). The observations that led to the conclusions concerning the differential expression of CALPS in *A. deanei* wild and aposymbiotic strains depicted from Fig. 1 and in Tables 1 and 2 are summarized in Table 3.

**Table 3:**
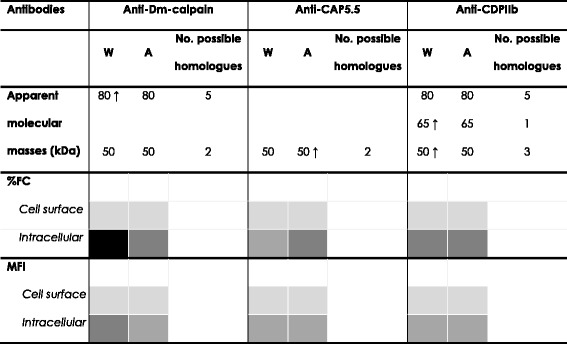
Differential expression of calpain-like proteins in *Angomonas deanei* according to the results presented in Fig. 1 and Tables 1 and 2.

Symbols used in this table: W, wild type strain; A, aposymbiotic strain; **↑**, proteins with a significant higher expression (*P <*0.05) in the marked strain; % FC, percentage of fluorescent cells and MFI, mean of fluorescence intensity, both in flow cytometry analysis. The grey scale indicates in % FC: less than 10 % (light grey); 10-20 % (medium grey), approximately 40 % (dark grey) and more than 80 % (black). In MFI levels, the grey scale indicates: less than 10 (light grey), 10–20 (medium grey) and approximately 40 (dark grey)

Although CALPs were more abundantly detected in the intracellular environment, membrane labeling is an important issue. The possible membrane targeting of some of these CALPs in *A. deanei* wild type and aposymbiotic strains was already suggested by the presence of potential acylation sites in these proteins (Table 1). In this sense, a previous work from our group using the same panel of antibodies raised against different calpains has shown, by flow cytometry, the binding of these antibodies to the surface of epimastigote forms of *T. cruzi* Y strain [18]. The blockage of CALPs by the pre-treatment of epimastigotes with anti-Dm-calpain antibody led to a significant reduction on the capacity of adhesion to its invertebrate vector (*Rhodnius prolixus*) gut in a dose-dependent manner [18], which points to a potential function of CALPs in this parasite.

### Effects of calpain inhibitors on *A. deanei* wild type and aposymbiotic strains growth rate

**Fig. 2:**
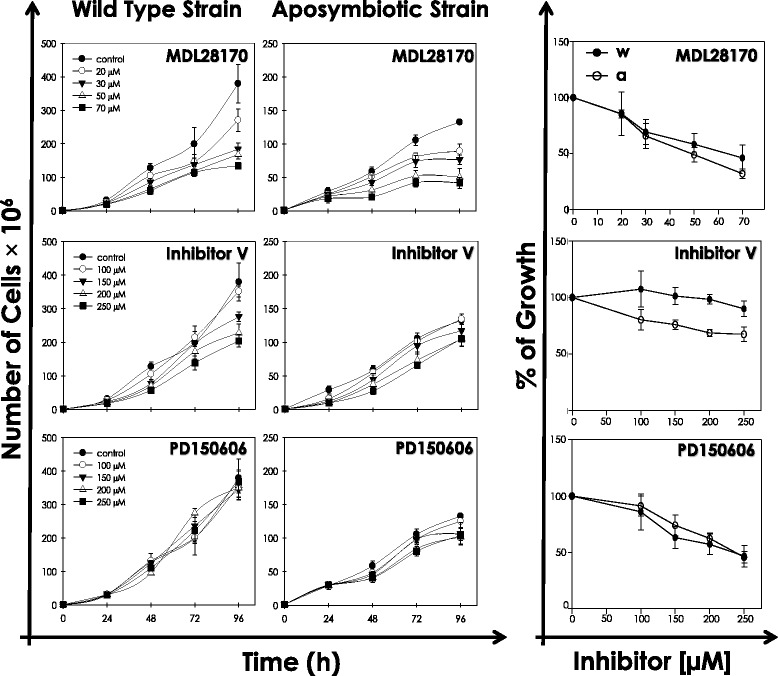
Effect of calpain inhibitors on the growth rate of *Angomonas deanei* wild type *(w)* and aposymbiotic *(a)* strains. *At left,* the growth pattern was followed in the absence (control) and the presence of MDL28170, PD150606 and calpain inhibitor V for 96 h. MDL28170 was assayed in concentrations ranging from 20 to 70 μM, and PD150606 and calpain inhibitor V were tested in concentrations ranging from 100 to 250 μM. Each inhibitor was added to the cultures at 0 h, and viable cells were counted daily by Trypan blue exclusion and mobility in a Neubauer chamber. DMSO, used as the drugs diluent, did not interfere on the growth behavior. The different ratio scales used to express the growth rates of each strain were necessary due to the lowest values obtained for the aposymbiotic strain. *At right,* the growth pattern was expressed as percentage of growth to each calpain inhibitor in the same concentrations cited above after 48 h. Data shown are the mean ± standard deviation of three independent experiments performed in triplicate

The effect of calpain inhibitors on the growth rate of wild type and aposymbiotic strains of *A. deanei* was approached by the incubation of cells in the absence and presence of different concentrations of each compound, and cell growth was then monitored for four days by counting the viable parasites in a Neubauer chamber. At first, calpain inhibitors were added to *A. deanei* cells at concentrations ranging from 20 to 70 μM. The results showed that growth of both strains was reduced in the presence of the reversible, competitive inhibitor MDL28170 in a dose-dependent manner (Fig. 2). The IC_50_ value determined after 48 h of cultivation was 64.4 μM for the wild type strain and 51.3 μM for the aposymbiotic strain.

Previous studies from our group demonstrated that MDL28170 was able to reduce the proliferation of *L. amazonensis* promastigotes [16] and *T. cruzi* epimastigotes [17] in a dose-dependent manner, with IC_50_ values of 19 μM and 31.7 μM, respectively. The same inhibitor was also able to reduce the viability of infective, non-replicative trypomastigote forms of *T. cruzi*, displaying a LD_50_ value of 20.4 μM after 24 h [28]. Further studies showed that MDL28170 was able to inhibit the interaction of trypomastigotes with macrophages and intracellular survival [28], besides interfering in the parasite adhesion to the insect midgut and in the differentiation process of epimastigotes into metacyclic trypomastigotes [18]. Interestingly, in *L. amazonensis*, MDL28170 was also shown to induce the expression of apoptotic markers [29].

In a comparative analysis of the effect of this drug in *A. deanei*, our results showed evidence towards the occurrence of decreased susceptibility of both wild type and aposymbiotic strains in comparison to those pathogenic species. It is interesting to point out that calpain-like cysteine peptidases constitute the largest gene family identified in the *A. deanei* (85 members) genome [15], even in higher numbers when compared to the abundant gene family in *T. brucei* (18 members), *T. cruzi* (24 members) and *Leishmania* spp. (27 members) [13]. A higher capacity was also observed against the aposymbiotic strain in comparison to the wild type strain, which may reflect the differences in metabolism caused by the lack of the endosymbiont. In this sense, biochemical and genomic studies revealed that most amino acid biosynthetic routes are in the symbiont genome and then complete essential metabolic pathways of the host protozoan [5, 6, 15].

Human calpains are inhibited by the regulatory endogenous protein calpastatin [30], but the lack of availability of isoform-specific calpain inhibitors has hindered elucidation of their exact physiological roles [10]. Although MDL28170 is considered a relatively specific calpain inhibitor, its action to a lesser extent against cysteine peptidases other than calpain, such as cathepsin B, cannot be ruled out due to the similarity of the active site among different classes of cysteine peptidases [31]. This compound is just one of the available calpain inhibitors screened for several human diseases that are believed to be calpain-associated pathological disorders [32, 33]. In this context, the action of two additional calpain inhibitors was investigated against *A. deanei* wild type and aposymbiotic strains, since to the best of our knowledge no such study has been reported in trypanosomatids: the non-competitive calpain inhibitor PD150606 and the irreversible, competitive calpain inhibitor V.

When compared to MDL28170, the calpain inhibitors V and PD150606 did not affect the parasite proliferation in both strains tested up to 70 μM (data not shown). After this preliminary screen, both inhibitors were tested at concentrations ranging from 100 to 250 μM. The calpain inhibitor PD150606 impaired multiplication of both strains only at concentrations higher than 150 μM after 48 h of cultivation (Fig. 2), and the IC_50_ value was calculated as 231.6 μM and 248.3 μM for the wild type and aposymbiotic strains, respectively, which is in contrast to the highest sensitivity of the aposymbiotic strain to MDL28170. Conversely, the calpain inhibitor V at the highest concentration used only marginally diminished the proliferation of both strains (Fig. 2). Cells cultured in the presence of DMSO (vehicle of the three calpain inhibitors) at the dose used to dissolve the highest concentration of each calpain inhibitor did not present any significant alteration on the growth pattern (Fig. 2). In addition, the anti-trypanosomatid activity of MDL28170 and PD150606 was reversible, since cells pre-treated for 48 h with the calpain inhibitors at the IC_50_ values resumed growth when subcultured in drug-free fresh medium (data not shown).

Differences in the degree of inhibition of calpain activity might be explained by differences in the chemical structure, mechanisms of action or specificity of calpain inhibitors for a particular calpain structure [32–34], which is an important issue, especially for invertebrates and lower eukaryotes displaying non-typical calpains, many of them probably with no proteolytic activity. However, even not displaying proteolytic activity, the detection of their expression may point to organism-specific functions for these proteins [10], and it has been speculated that calpains devoid of enzymatic activity are involved in regulatory processes [13]. PD150606 selectively inhibits mammalian calpains relative to other peptidases, such as cathepsin B and cathepsin L, since it targets the calcium-binding domains in both calpain subunits that are essential for enzymatic activity and not found in cathepsins [35]. However, these calcium-binding motifs are absent in trypanosomatid CALPs, although amino acid residues that are critical for binding of calcium in mammalian calpains are partially conserved in some kinetoplastid sequences [13]. In addition, it was recently proven that PD150606 must be also acting at a site on the peptidase core domain of calpains to perform its inhibition [36]. These aspects could explain the discrepancy between the inhibitory effects of MDL28170 and PD150606 in *A. deanei* growth. The lack of growth inhibition in the presence of calpain inhibitor V, even at the highest concentration used, could be explained by the absence of a significant inhibition of *A. deanei* proteins/peptidases or by a poor cell membrane permeability of the latter. The answer to this question demands further experiments in order to demonstrate the degree of cell-penetrability of inhibitor V, as previously confirmed to MDL28170 [31] and PD150606 [35].

The biological functions of CALPs in trypanosomatids, most of them probably with no proteolytic activity [13], have not been fully described. A study in *T. cruzi* epimastigotes found a correlation between induced stress and increased expression of a specific CALP [37], while a proteomic analysis in benznidazol-resistant *T. cruzi* epimastigotes revealed the superexpression of a particular CALP [38]. In *Leishmania donovani,* the expression of a specific CALP was correlated to drug-induced programmed cell death and the modulation of susceptibility to antimonial drugs [39]. In addition, life-cycle specific expression of CALPs was demonstrated in *Leishmania major* [40], and depletion of life-cycle specific CALPs in *T. brucei* interferes with cytokinesis [41]. All these data point to the importance of the expression of distinct CALPs in many aspects of the parasites’ metabolism.

The results presented in this work raised the question as to whether CALPs expression may be influenced by the presence of the endosymbiont. Comparative studies between symbiont-harboring trypanosomatids (wild type and aposymbiotic strains) and trypanosomatid species that do not harbor endosymbionts have permitted inferences about the symbiont dependence and contribution in the overall metabolism [5]. The available data indicate that the presence of the endosymbiont induces morphological and biochemical changes in the host trypanosomatid [5, 6]. Analyses of the essential amino acid pathways revealed that most biosynthetic routes are in the symbiont genome [6]. The symbiotic bacterium also preserved genes which code enzymes that complete essential metabolic pathways of the host trypanosomatid, such as heme and vitamin production [42, 43]. In addition, the endosymbiont actively contribute to the metabolism of the trypanosomatid host by enhancing phospholipid production [44]. As a result, symbiont-harboring trypanosomatids present low nutritional requirements when compared to other species of the Trypanosomatidae family [5].

Furthermore, studies of cellular interaction showed that endosymbiont-bearing cells interact better with insect cell lines and explanted guts, when compared with their aposymbiotic counterpart strains [45]. This seems to occur because *A. deanei* aposymbiotic strain differ from the respective wild type strain in the amount of their surface glycoconjugates and proteolytic enzymes, including gp63-like metallopeptidase [7, 46, 47], which influences the protozoan interaction with the insect host. The metallopeptidase gp63, a major glycoprotein found in the leishmanial cell surface, is also present in *A. deanei* [26]. This protein mediates the adhesive process of the protozoan to *Aedes aegypti* explanted guts, as demonstrated using anti-gp63 antibodies. The higher expression of gp63-like molecules on the surface of symbiont-bearing trypanosomatids, when compared to the aposymbiotic cells, may be correlated with a more efficient interaction of the wild strain with insect guts [46]. The role of gp63-like molecules in such interactions may have caused the expansion of this gene family in endosymbiont-bearing organisms [15].

*A. deanei* also contains a large number of genes for calpain-like molecules in comparison to other trypanosomatids, reflecting adaptations to the presence of the symbiont [15], which is paralleled by the absence of calpain molecules in the symbiont. In this association between the protozoan and the bacterium, the endosymbiont is unable to survive and replicate once isolated from the host. The host protozoan provides a stable environment to the symbiont, and some evidence indicates that the endosymbiont exploits the trypanosomatid’s energy metabolism by utilizing the ATP generated by host glycosomes, which are organelles that compartmentalize glycolytic enzymes [5]. The synchrony in cellular division is another striking feature of this symbiotic relationship. Typically, there is one symbiont per cell, which implies that the symbiont and host cell divide synchronously [5]. Recently, Catta-Preta and coworkers [48] provided evidence that symbiont segregation is dependent on the progression of the protozoan cell division cycle, including nuclear mitosis and/or microtubule organization, indicating that the host trypanosomatid exerts tight control over the bacterial cell number. Simultaneoulsy, the presence of the endosymbiont causes ultrastructural alterations in the host trypanosomatid, such as a reduced paraflagellar structure [5].

Taken together, these data may indicate that the relatively large amount of calpain-like genes detected in *A. deanei* [15] could be related to the presence of the endosymbiont, which would require the higher expression of CALPs due to a more complex regulation of the cell cycle and intracellular organelle distribution, as cytosolic calpains were found to regulate cytoskeletal remodeling, signal transduction and cell differentiation [10, 12, 15]. This fact may be correlated to the higher expression of the majority of CALPs detected in the present study in the wild strain than in the aposymbiotic strain; in parallel, it could explain the higher sensitivity of the aposymbiotic strain to the calpain inhibitor MDL28170 than the wild strain. The results presented in this study may contribute to the investigation of the existence and functions of such a variety of CALPs in trypanosomatids.

## Conclusions

In recent years, it has become obvious that calpains and CALPs play vital roles not only in mammalian cells but in invertebrates and distinct microrganisms as well. The results described in the present paper indicate that calpain-like molecules, the largest gene family identified in *A. deanei* genome, had their expression possibly influenced by the presence of the endosymbiont. The usage of anti-calpain antibodies with different specificities and the flow cytometry and Western blotting techniques allowed the detection of distinct CALPs. In addition, treatment with the calpain inhibitor MDL28170 affected efficiently the growth rate of both the wild type and the aposymbiotic strains, with a higher inhibitory capacity against the latter, which may indicate metabolic differences caused by the lack of the symbiont. However, the susceptibility of both strains was decreased when compared to the pathogenic species *T. cruzi* and *L. amazonensis.* As a whole, the better understanding of the expression of such an important group of proteins in trypanosomatids is crucial to explain the roles they should display in the physiology of these microrganisms.
